# Sorting Nexin 9 Recruits Clathrin Heavy Chain to the Mitotic Spindle for Chromosome Alignment and Segregation

**DOI:** 10.1371/journal.pone.0068387

**Published:** 2013-07-05

**Authors:** Maggie P. C. Ma, Phillip J. Robinson, Megan Chircop

**Affiliations:** Children’s Medical Research Institute, the University of Sydney, Westmead, New South Wales, Australia; Florida State University, United States of America

## Abstract

Sorting nexin 9 (SNX9) and clathrin heavy chain (CHC) each have roles in mitosis during metaphase. Since the two proteins directly interact for their other cellular function in endocytosis we investigated whether they also interact for metaphase and operate on the same pathway. We report that SNX9 and CHC functionally interact during metaphase in a specific molecular pathway that contributes to stabilization of mitotic spindle kinetochore (K)-fibres for chromosome alignment and segregation. This function is independent of their endocytic role. SNX9 residues in the clathrin-binding low complexity domain are required for CHC association and for targeting both CHC and transforming acidic coiled-coil protein 3 (TACC3) to the mitotic spindle. Mutation of these sites to serine increases the metaphase plate width, indicating inefficient chromosome congression. Therefore SNX9 and CHC function in the same molecular pathway for chromosome alignment and segregation, which is dependent on their direct association.

## Introduction

Precise organization of a mitotic spindle is an important part of accurate chromosome alignment and segregation during cell division [1,2]. In higher organisms discrete bundles of microtubules (MTs), termed kinetochore fibers (K-fibers), extend from the spindle poles and attach to the kinetochores of chromosomes to assist chromosome alignment at the metaphase plate [3]. K-fibers are cross-linked by inter-MT bridges contributing to mitotic spindle stabilization during chromosome movement [3]. Clathrin is responsible for these bridges [4].

Clathrin is a triskelion structure consisting of three heavy chains (CHC; ~190 kDa) each with an associated clathrin light chain (CLC; ~25 kDa) [5]. During interphase, clathrin participates in membrane trafficking by coating membranes for vesicular transport [6]. During mitosis, membrane trafficking is reduced [7] and CHC assumes a new role. It localizes to the mitotic spindle due to the N-termini of each of the three CHC molecules binding to the K-fibers [4,8]. Thus clathrin forms a structural bridge cross-linking the K-fibres and stabilizes the mitotic spindle [4]. CHC action at the mitotic spindle is independent of its endocytic function. CHC is unable to directly bind MTs and once it arrives at the mitotic spindle it is tethered there by an as yet unidentified binding partner [8].

Sorting nexin 9 (SNX9) is a major binding partner for CHC during clathrin-mediated endocytosis (CME) [9]. SNX9 belongs to the sorting nexin (SNX) superfamily of proteins which have a SNX-phox-homology (PX) domain that allows association with a range of phosphoinositides [10]. SNX9, SNX18 and SNX33 belong to the SNX9 subfamily, which share an Src-homology 3 (SH3) domain that allows interaction with proline-rich proteins [11] and a Bin-Amphiphysin-Rvs (BAR) domain for dimerisation that contributes to shaping membrane curvature [12]. The SNX9 subfamily members play critical roles in endocytosis [9,13,14]. Both SNX33 [15] and SNX9 [16] also have roles in mitosis, but at different points in the cycle. Both function during cytokinesis, but SNX9 also functions during metaphase. Like CHC, the metaphase role of SNX9 is not dependent on its endocytic function [16] while the other members of the SNX9 subfamily are not required for metaphase [4,16]. SNX9 and CHC directly interact via the low complexity (LC) domain of SNX9 [9], raising the possibility that they may also function together at the mitotic spindle. Our aim was to determine if SNX9 and CHC function co-operatively in the same or different molecular pathways during metaphase.

## Materials and Methods

### Cell culture and transfection

HeLa human cervical carcinoma cells were maintained in RPMI 1640 medium supplemented with 10% foetal bovine serum (FBS). Cells were grown at 37°C in a humidified 5% CO_2_ atmosphere. Cells were seeded at 50-60% confluence (1 × 10^5^ cells per 10 cm dish, 0.5× 10^5^ cells per well of a 6-well plate; 0.2 × 10^5^ cells per well of a 12-well plate). For siRNA analyses, cells were transfected with 1000 pmol of siRNA (per 10 cm dish for immunoblotting), 200 pmol of siRNA (per well of a 6-well plate for immunofluorescence and time-lapse microscopy experiments) or 100 pmol (per well of a 12-well plate for immunofluorescence and time-lapse microscopy experiments). For DNA transfections, 1.5 µg of the indicated plasmid DNA was used in each well of a 6-well plate. Cells were transfected with Lipofectamine 2000 (Invitrogen) according to the manufacturer’s instructions.

### Cell synchronization

HeLa cells grown on glass coverslips were synchronized at the G_2_/M border by treatment with the selective Cdk1 small molecule inhibitor RO-3306 (9 µM) for at least 18 h. Cells were allowed to progress through mitosis upon RO-3306 wash out. Following RO-3306 wash out, cells were incubated at 37°C/5% CO_2_ for 80 min (metaphase), 110 min (anaphase) and 140 min (cytokinesis) as previously reported [16–18].

#### Plasmid constructions

GST-SNX9 and GFP-SNX9 were provided by Prof. Sandra Schmid [19]. Point mutations of GST-SNX9 and GFP-SNX9 (LC1, LC1W1, LC2, LC2W1) were generated using the QuikChange site-directed mutagenesis kit (Stratagene). The siRNA target sequences in the sense orientation for the following proteins are: CHC: 5’- GCAAUGAGCUGUUUGAAGA-3’ [20]; SNX9: 5’-AACCUACUAACACUAAUCGAU-3’ [21]; SNX18: 5’-CGUCAUGGACCUAUUAGCGCUGUAU-3’; SNX33: 5’-CAAGAUCGCUGAGACAUACUCCA-3’; Luciferase: 5’-CGUACGCGGAAUACUUCGA-3’.

#### Time-lapse microscopy

Immediately following release into the cell cycle G_2_/M synchronized cells were viewed with an Olympus IX81 inverted microscope and a time-lapse series was acquired using a fully motorized stage, 10× objective, and Metamorph software using the time-lapse modules [17,18]. Temperature control was achieved using the Incubator XL, providing a humidified atmosphere with 5% CO_2_. Imaging was performed for 20 h with a lapse time of 10 min.

### Immunofluorescence microscopy

Cells were fixed in ice-cold 100% methanol for 3 min at -20 ^°^C and blocked in 3% bovine serum albumin (BSA)/PBS for 45 min prior to incubation with the required primary antibody Antibodies used for immunofluorescence microscopy analysis included: anti-SH3PX1 (anti-SNX9, NB100-2813, Novus Biological), anti-SNX18 (GTX106319, GeneTex), anti-SH3PX3 (anti-SNX33, H00257364-D01P, Abnova), anti-CHC (BD Transduction, 610500), anti-Aurora A (Cell Signalling, 4718), anti-phospho Aurora A (Thr-288) (Cell Signalling, 3079), anti-TACC3 (Santa Cruz, sc-22773), anti-HURP (Abcam, ab84509). Fluorescein- or Texas Red dye-conjugated AffiniPure secondary antibodies (Jackson ImmunoResearch Laboratories, Inc.) were then applied. Cell nuclei were counterstained with DAPI (4’, 6’-diamidino-2-phenylindole; Sigma). Cells were washed three times with PBS between each step except for after blocking and viewed and scored with a fluorescence microscope (Olympus IX80).

### Image acquisition and analysis

Fluorescence images were captured under an Olympus IX81 inverted microscope using 60 × or 100 × oil immersion lenses. A Z-stack of each cell was acquired where 31 z-sections, each 0.2 µm thick, were obtained (total thickness of the Z-stack = 6 µm). Images were deconvolved using AutoDeblur v 9.3 (AutoQuant Imaging, Watervliet, NY) and a maximum projection image was obtained. For fluorescence intensity quantification, the regions of interest were marked and the stack integrated intensities of the regions were measured using Metamorph software (Version 7.7.0.0.).

### Pull-down binding assay

HeLa cells lysates were prepared as described previously [22]. In brief, cells were collected by centrifugation, washed with PBS, then resuspended in ice-cold lysis buffer for sonication [25 mM Tris-HCl pH 7.4, 150 mM NaCl, 1 mM EDTA, 1 mM EGTA, 1 mM PMSF, 1% Triton X-100, and EDTA-free Complete protease inhibitor cocktail (Roche)] followed by incubation on ice for 30 min. The supernatant was collected following centrifugation at 13,000 rpm for 30 min at 4°C. Pull-down experiments were performed by incubating the GST-SNX9 LC1, LC2, LC1W1 and LC2W1 mutants bound to GSH-Sepharose beads with 2 mg of HeLa cell lysates for 1 h at 4°C. Beads were washed extensively with ice-cold lysis buffer [23]. Proteins bound to the beads were eluted by heat incubation at 85 °C for 5 min. Proteins (10 µl) were resolved on a 7.5-15% gradient SDS-PAGE gel and stained with Colloidal Coomassie Blue stain. In parallel, 40 µl of each sample was resolved on a 7.5-15% gradient SDS-PAGE gel for Western blot analyses.

### Immunoblotting

Cell lysates (200 µg) were fractionated by SDS-PAGE for immunoblot analysis with the following antibodies: anti-SNX9 (Santa Cruz), anti-SNX18 (GeneTex), anti-SNX33 (a gift from Prof. Stefan F. Lichtenthaler [24];, anti-CHC (BD Transduction, 610500), anti-TACC3 (Santa Cruz, sc-22773), anti-β-actin (Sigma, A3854), anti-dynI phospho-S778 which detects dynII phospho-S764 [25], anti-dynamin II (Santa Cruz, sc-6400). Antibody bound to the indicated protein was detected by incubation with a horseradish peroxidase-conjugated secondary antibody (Jackson ImmunoResearch Laboratories, Inc.). Blotted proteins were visualized using the ECL detection system (Pierce).

### Microtubule Nucleation Assay

The *in vivo* microtubule nucleation assay was performed as previously described with modification [26]. Metaphase-synchronised HeLa cells were placed on ice for 1 h to depolymerise microtubules. The ice-cold medium was aspirated and medium pre-warmed to 37 ^°^C was added for 1, 3 or 10 min. Cells that were not released into the pre-warmed medium were also collected as a control (0 min) to ensure microtubules were completely depolymerised. Cells were washed with ice-cold PBS then fixed in ice-cold 100% methanol for 3 min at -20 ^°^C before being processed for immunofluorescence microscopy by staining for α-tubulin and γ-tubulin to visualize microtubule regrowth and the spindle poles, respectively. The length of the microtubules was measured using Metamorph software (Version 7.7.0.0.).

### Endocytosis Assay

Quantitative high-throughput receptor-mediated endocytosis (RME) assays were performed as previously described [27,28] using Transferrin (Tfn) conjugated to Texas Red in untreated and siRNA transfected HeLa cells for 10 min. In brief, HeLa cells were grown in fibronectin-coated (5 µg/mL) 96-well plates. The cells were serum-starved overnight (16 h) in DMEM without FCS then incubated for 30 min with 4 µg/ml of Texas Red-Tfn for 8 min at 37°C. Cell surface-bound Texas Red-Tfn was removed by an ice-cold acid wash (0.2 M acetic acid + 0.5 M NaCl, pH 2.8) for 10 min then rinsed with ice-cold PBS for 5 min. Cells were then fixed with ice-cold 4% PFA in PBS for 15 min and processed for immunofluorescence microscopy analysis as described above. Quantitative analysis of the inhibition of Tfn endocytosis in HeLa cells was performed on transfected cells by an automated acquisition and analysis system (Image Xpress Micro, Molecular Devices, Sunnyvale, CA). Average cell: vesicle integrated intensity of the Tfn signal/cell was calculated using the IXM software and expressed as a percentage of control cells. Data was analyzed using Prism 5 (GraphPad Software Inc.) and expressed as mean ± s.e.m for triplicates.

## Results

### Depletion of SNX9 and CHC delay mitotic progression at two points

**Figure 1:**
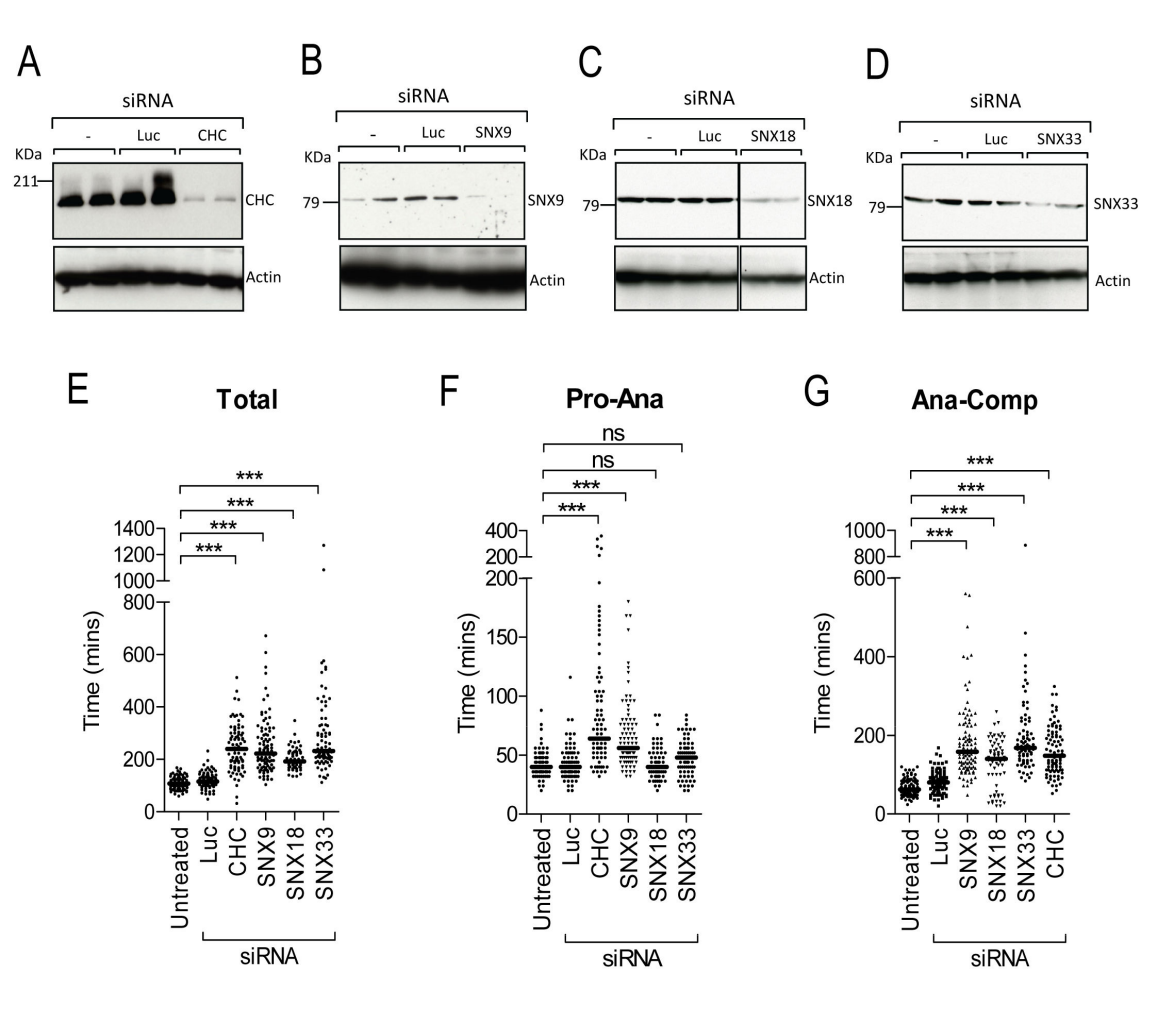
SNX9 depletion phenocopies CHC depletion during mitosis. HeLa cells were either untreated (-), or transfected with luciferase siRNA (Luc) or siRNA targeting CHC (A), SNX9 (B), SNX18(C) and SNX33 (D). At 72 h post-transfection, protein lysates (200µg) were immunoblotted with anti-CHC, anti-SNX9, anti-SNX18 and anti-SNX33 antibodies respectively. Actin was used as a loading control. (E–G) HeLa cells were treated with siRNA targeting Luc, CHC, SNX9, SNX18 and SNX33 and visualised by time-lapse microscopy. Graphs show the time that each individual cell took to undergo mitosis (n > 100 per sample; E) as well as the following mitotic transitions: prophase to anaphase (F), and anaphase to completion (G). Pro, prophase; Ana, anaphase; Comp, completion. Data shown in E-G are from one representative experiment. Similar results were obtained in at least two independent experiments. The median time per sample is represented by a solid black line. ***, *p* <0.001 (One-way ANOVA).

To ask if CHC and SNX9 functionally co-operate during metaphase we compared the effect of siRNA depleting CHC, SNX9, SNX18 and SNX33 on mitotic progression of HeLa cells. Our previous characterisation of the SNX siRNAs have shown that they have no off-target action towards other SNX proteins and their mitotic induced phenotypes can be rescued by overexpressing the relevant SNX protein [16]. Consistent with our previous report, at 72 h post-transfection, immunoblots revealed a satisfactory knockdown of all four proteins (Figure 1A–D). Depletion of any of the four caused cells to spend significantly longer time in mitosis (Figure 1E) as previously reported [4,16]. By assessing the time that depleted cells took to progress from prophase (mitotic entry and nuclear envelope breakdown) to anaphase (chromosome segregation) we found that CHC and SNX9, but not SNX18 or SNX33, showed significantly delayed transition, thus they have earlier stage roles (Figure 1F). The analogous phenotypes produced by depletion of CHC and SNX9 are of a similar extent. In contrast, all four endocytic proteins were required for efficient progression through the final stages of mitosis from anaphase to cytokinesis completion (Figure 1G). This is consistent with previous reports [16,29,30] and the concept that endocytosis may be required for the final stages of cytokinesis [7]. In contrast, endocytosis is blocked during earlier mitotic stages [7]. These observations suggest that SNX9 and CHC might functionally co-operate during metaphase.

### Depletion of CHC and SNX9 disrupts MT nucleation and K-fibers

**Figure 2:**
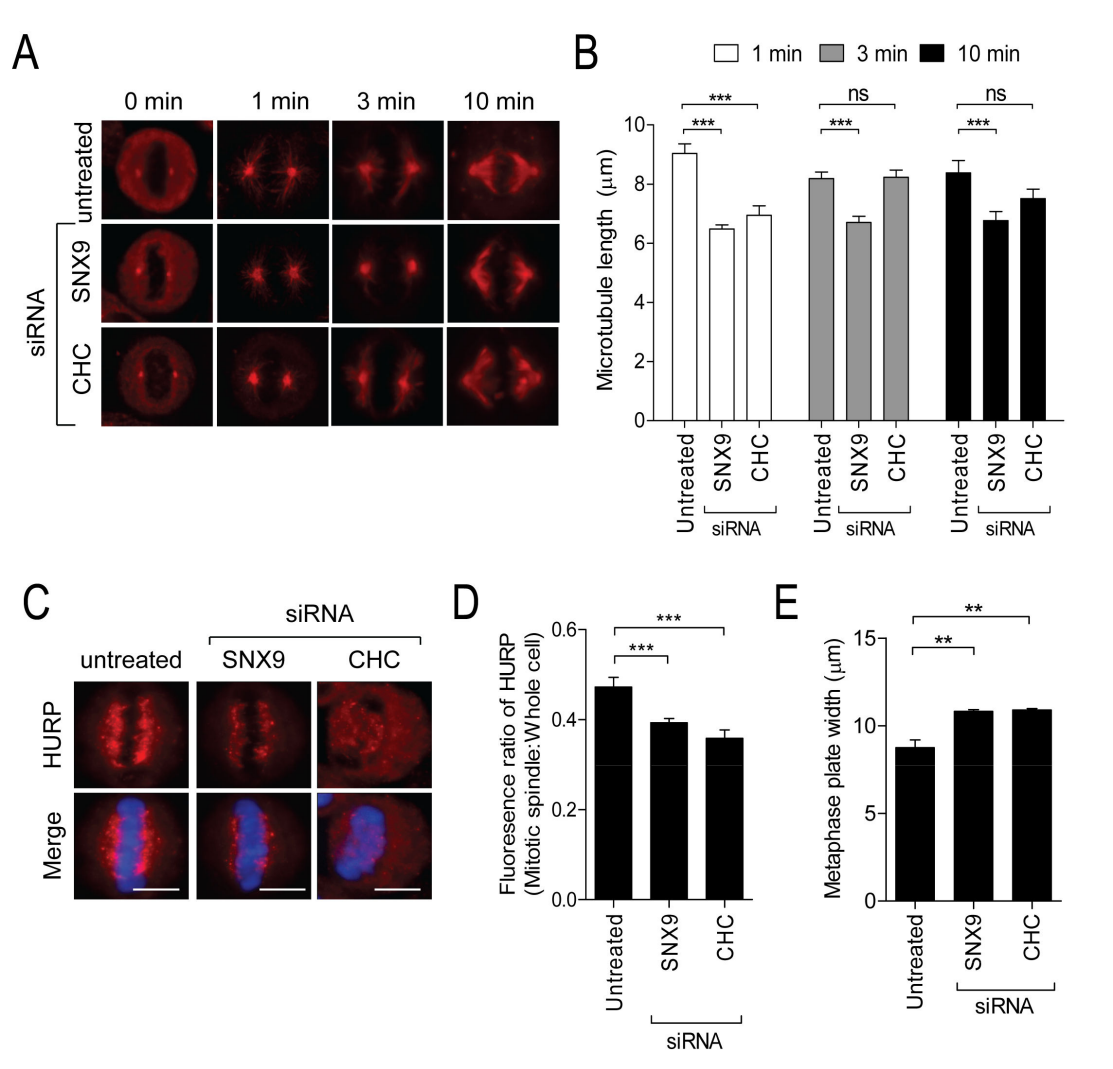
SNX9 and CHC are required for MT nucleation and K-fibre organization at the mitotic spindle. (A–B) Untreated HeLa cells and HeLa cells transfected with SNX9 or CHC siRNA, were subjected to a MT nucleation assay as described in methods section. Representative microscopy images illustrating MT regrowth from the spindle poles after the time period (A). Graphs show the length of the longest MT (mean ± S.E.M.) at each time point (n=11-19 cells per sample from two independent experiments; B). (C) Representative microscopy images of a HURP stained untreated, SNX9-depleted and CHC-depleted HeLa cell in metaphase, to highlight K-fibres (upper panels). DNA shown in lower panels. (D) The graph represents the fluorescence intensity ratio of HURP at the mitotic spindle over the whole cell. Scale bars represent 10 µm. ns, not significant; **, *p* < 0.01; ***, *p* < 0.001 (One-way ANOVA). (E) The graph (mean ± S.E.M. from three independent experiments) shows the width of the centre of the metaphase plate of SNX9 and CHC-depleted HeLa cells compared to that in untreated HeLa cells in metaphase.

To test whether SNX9 and CHC function in the same role during metaphase we compared the metaphase failure phenotypes of depleted cells, based on previous observations that CHC contributes to mitotic spindle assembly and stability through MT nucleation and K-fibre stability, respectively [4,31]. An *in vivo* MT regrowth assay revealed that like CHC, SNX9 is required for MT nucleation, since the regrowth of MT asters was significantly disrupted in SNX9-depleted cells at 1 and 3 min post-recovery (Figure 2A-B). MT asters in CHC-depleted cells had regrown to a similar extent as those in untreated cells after 10 min. In contrast, MT regrowth remained impaired in SNX9-depleted cells at this time. We next measured the fluorescence intensity at the mitotic spindle of the K-fibre marker, hepatoma up-regulated protein (HURP). HURP staining was significantly reduced at the mitotic spindle of both SNX9- and CHC-depleted metaphase cells (Figure 2C-D). Therefore both CHC and SNX9 are involved in stabilising K-fibres. Depletion of SNX9 and of CHC both caused a significant increase in the width of the metaphase plate (Figure 2C & E), an indicator of chromosome alignment. These observations support the idea that both proteins participate in the mechanisms that establish and stabilise the mitotic spindle.

### SNX9 is involved in efficient recruitment of CHC and TACC3, but not Aurora A, to the mitotic spindle

**Figure 3:**
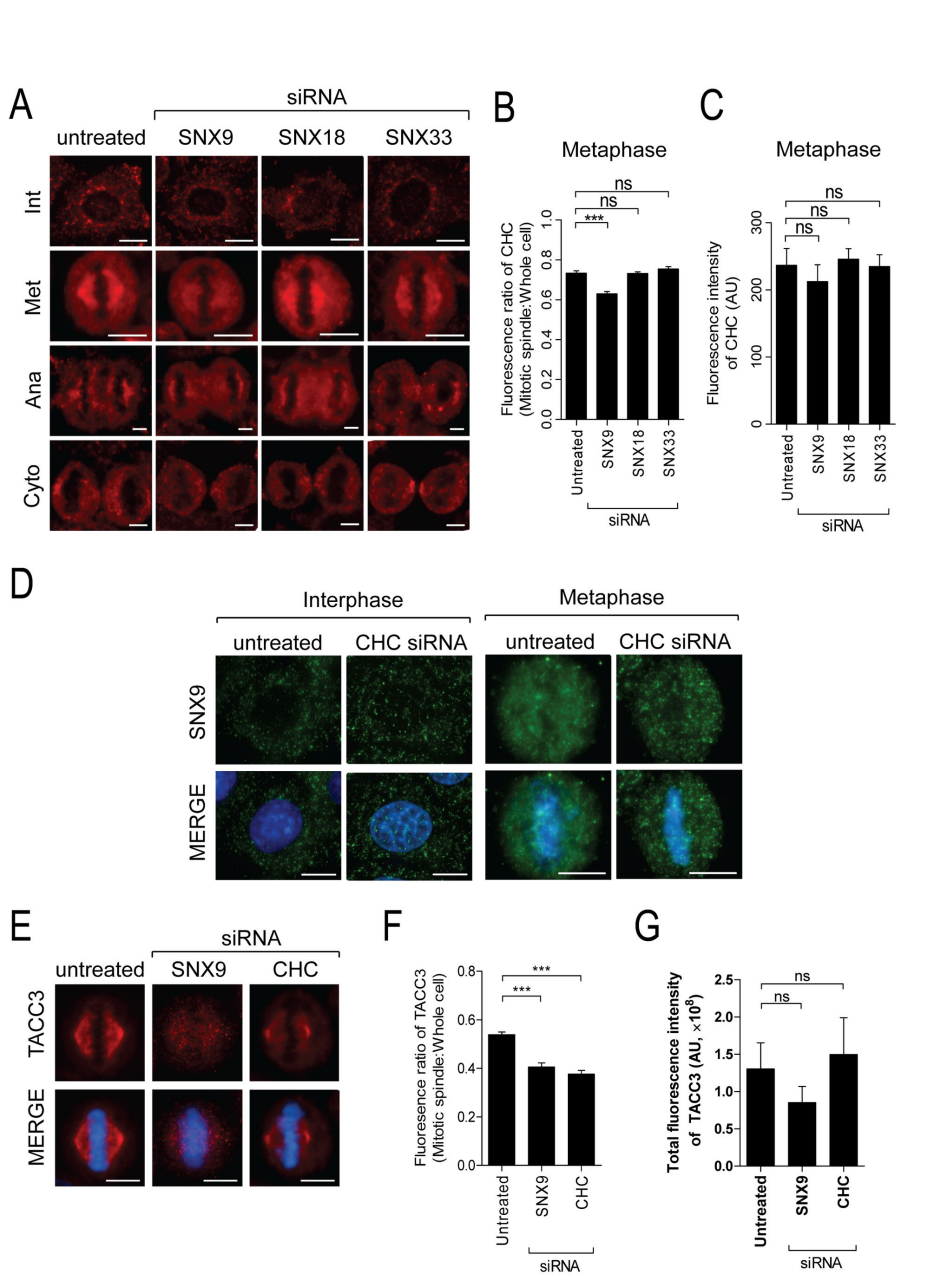
SNX9 is required for efficient recruitment of CHC and TACC3 to the mitotic spindle. (A) Representative microscopy images illustrating the localization of CHC during interphase (Int) and the indicated mitotic stages in untreated cells and cells depleted of SNX9, SNX18 or SNX33 by siRNA. Met, metaphase. Ana, anaphase. Cyto, cytokinesis. Scale bars represent 10 µm. (B–C) The graphs represents the fluorescence intensity ratio of CHC at the mitotic spindle over the whole cell (B) and the overall fluorescence intensity of CHC within the whole cell (C) during metaphase (mean ± S.E.M., n > 16 per sample). ns, not significant; ***, *p* < 0.001 (One-way ANOVA). (D) Representative microscopy images demonstrating the localization of SNX9 (upper panels) in untreated and CHC-depleted HeLa cells during interphase, and metaphase. DNA shown in lower panels. Scale bars represent 10 µm. (E–F) Untreated, SNX9 and CHC-depleted metaphase HeLa cells were stained for TACC3. Representative microscopy images of TACC3 localization (upper panels) in these cells are shown in D. DNA shown in lower panels. Graphs represents the amount of TACC3 in each cell, expressed as (F) ratio of fluorescence intensity at the mitotic spindle compared to the whole cell and (G) the overall fluorescence intensity of TACC3 within the whole cell (mean ± S.E.M., n = > 6).

CHC metaphase function utilises a signalling pathway involving Aurora A kinase and TACC3. Aurora A phosphorylates TACC3 at S558 upon mitotic entry, targeting it to the spindle poles [32] where phospho-TACC3 and ch-TOG form a complex with CHC to stabilise K-fibres [33,34]. Since SNX9 also localises to spindle poles [16] we asked whether it functions in the same pathway. We determined if SNX9 lies upstream of CHC by exploring whether it may be involved in recruiting it to the spindle. In untreated cells CHC was enriched at the spindle during metaphase (Figure 3A-B) as previously reported [4]. Fluorescence intensity measurements of CHC revealed that spindle enrichment was significantly reduced in SNX9-depleted metaphase cells and CHC was instead found in the cytoplasm. CHC mitotic spindle localization was reduced by 26% (Figure 3B) with a corresponding increase of 15.1% in the cytoplasm (determined by fluorescence intensity of CHC in whole cell minus fluorescence intensity of CHC on the spindle), but total CHC levels were unaffected (Figure 3C), indicating that SNX9 is involved in CHC recruitment. The role of SNX9 is specific since CHC localization and protein levels were not disrupted after SNX18- or SNX33-depletion (Figure 3A–C). The role of SNX9 in CHC function was limited to metaphase since the localization of CHC during interphase (Int) and later stages of mitosis (Ana and Cyto) was unaffected in cells depleted of SNX9, SNX18 or SNX33 (Figure 3A). Importantly, depletion of CHC did not disrupt the localization of SNX9 and phase measured (Figure 3D), indicating that SNX9 functions upstream of CHC during metaphase.

The order of recruitment of CHC and phospho-TACC3 at the spindle appears to involve CHC recruitment prior to that of TACC3 [31,35,36], although there is a recent contradictory report [8]. To assess the situation in our model system we determined the localization of TACC3 in CHC-depleted cells. We observe a significant reduction in TACC3 at the mitotic spindle in CHC-depleted cells (Figure 3E-F) in line with the original findings [31,35,36], and supporting the idea that CHC recruits TACC3 to the spindle in HeLa cells. We next assessed the role of SNX9 in TACC3 recruitment. SNX9 depletion significantly reduced TACC3 at the spindle (Figure 3E-F) to a similar level induced by CHC-depletion, with TACC3 distributing to the cytoplasm (Figure 3E). Therefore both CHC and SNX9 are involved in recruiting TACC3. SNX9 depletion had no effect on the metaphase localization of Aurora A and phospho-Aurora A (active form) at the mitotic spindle (Figure S1), suggesting that SNX9 functions downstream of this kinase. We also observed no effect on Aurora A or phospho-Aurora A localization in metaphase cells depleted of CHC or TACC3 (Figure S1), as previously reported [31]. The lack of contribution of SNX9, CHC and TACC3 to Aurora A mitotic localization or activation is consistent with Aurora A activity being required for mitotic entry [37]. Overall, our observations place SNX9 in the same molecular pathway as CHC, where it acts upstream of CHC and TACC3 to mediate spindle assembly and stability.

### The SNX9-CHC interaction is required for CHC and TACC3 mitotic spindle localization

**Figure 4:**
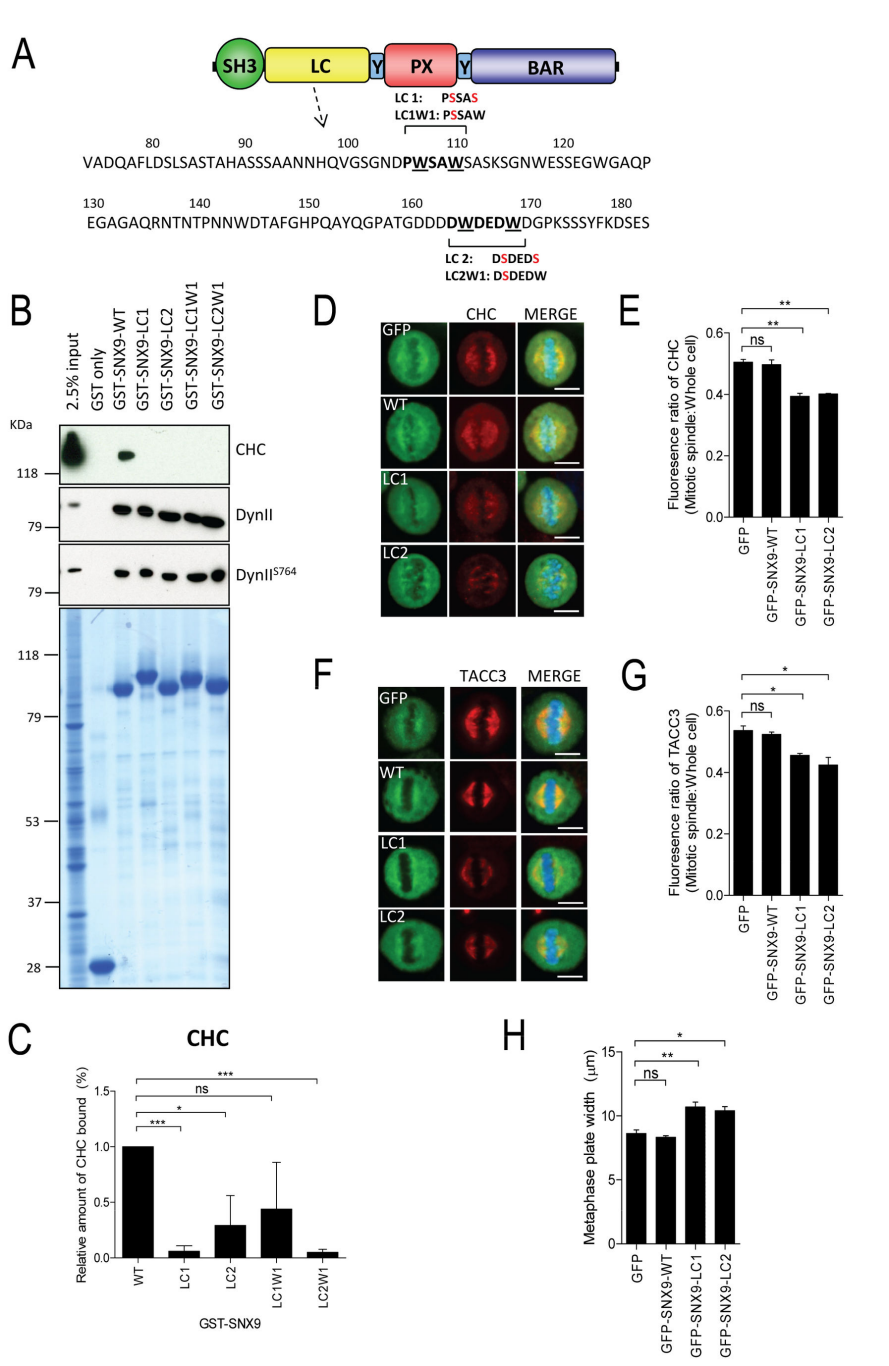
CHC interaction with the LC domain of SNX9 is required for efficient recruitment of mitotic spindle components. (A) A schematic diagram illustrating the domain structure of SNX9 and the amino acids in part of the low complexity sequence (LC domain). SNX9 contains an Src-homology 3 (SH3) domain at the N terminus followed by a low complexity (LC) domain, a phox-homology (PX) domain that is flanked by Yoke (Y) domains. A C-terminal Bin/Amphiphysin/Rvs (BAR) domain is located at the C-termini. Single and double mutations in LC1 (LC1 and LC1W1) and LC2 (LC2 and LC2W1) regions are shown whereby the tryptophan (W) residues (underlined) were mutated to serine (S). (B) GST alone, full length wild-type GST-SNX9 (WT) and GST-SNX9 harbouring LC1, LC1W1, LC2 and LC2W1 mutants coupled to glutathione-Sepharose were incubated with lysates from asynchronously growing HeLa cells and immunoblotted for CHC, dynII and dynII^S764^. Lower panel shows amount of GST fusion protein in each sample (10%) as determined by Coomassie Blue staining. Lysate (2.5%) was also immunoblotted for the above mentioned proteins to reveal input. (C) The amount of CHC bound to the GST-SNX9 proteins indicated in B were quantified by densitometry analyses of immunoblots. Graph illustrates the relative amount of CHC bound to mutant GST-SNX9 (mean ± S.E.M. from 3–4 independent experiments) compared to wild-type GST-SNX9. (D–G) Metaphase-synchronised HeLa cells expressing GFP alone or GFP-SNX9-WT, LC1 or LC2 mutants were stained for CHC and TACC3. Representative microscopy images of the localization of CHC (D) and TACC3 (F) in these cells are shown. Scale bars represent 10 µm. The graphs represent the fluorescence intensity ratio of CHC (E) and TACC3 (G) at the mitotic spindle over the whole cell. (H) The graph illustrates the width of the metaphase plate of those cells analysed in E and G. Data shown in graphs (E, G and H) represent mean ± S.E.M. from at least two independent experiments. n = 15-30 cells per sample in each experiment. ns, not significant; *, *p* < 0.05; **, *p* < 0.01 (One-way ANOVA).

We next asked whether the SNX9-CHC spindle recruitment involves their direct protein–protein interaction. Single and double mutations of W to serine (S) were generated for both _107_PWSAW (LC1: _107_PSSAS and LC1W1: _107_PSSAW) and _164_DWDEDW (LC2: _164_DSDEDS and LC2W1: _164_DSDEDW) sequences in GFP-SNX9 to disrupt CHC binding (Figure 4A). Pull-down experiments using asynchronously growing HeLa cells with wild-type and LC mutant GST-tagged SNX9 were performed (Figure 4B). All four LC mutants abolished SNX9 binding to CHC (Figure 4B-C), but did not disrupt its interaction with dynamin II (dynII, Figure 4B) or phospho-dynII (dynII^S764^) at the SH3 domain (Figure S2). This confirms that at least SNX9 W108 and W165 are required to directly bind CHC. A significant reduction in CHC (Figure 4D-E) and TACC3 (Figure 4F-G) enrichment at the mitotic spindle was observed in cells overexpressing any one of the four LC mutants. Consistent with the finding that SNX9-depletion impairs chromosome congression (Figure 2E), the LC mutants also caused a significant increase in the width of the metaphase plate (Figure 4H). Therefore CHC directly associates with the LC domain of SNX9 for recruitment of CHC and subsequently of TACC3 to the spindle.

### SNX9-LC mutants do not block clathrin-mediated endocytosis

The metaphase role of CHC is independent of its endocytic function [4]. Our observations thus far also suggest that the metaphase role of SNX9 is also independent of its endocytic function. To confirm this we examined the effect of the LC1 and LC2 mutants on the cellular uptake of Alexa-Fluor 594-conjugated Transferrin (Tfn), a classical marker for the CME pathway. Tfn uptake was unaffected in HeLa cells overexpressing GFP-SNX9-LC1 or GFP-SNX9-LC2 mutants (Figure S3), consistent with our previous finding that SNX9 depletion does not block Tfn uptake during metaphase [16]. Therefore the SNX9-CHC association at the spindle during metaphase is involved in an endocytosis-independent function.

## Discussion

We find that SNX9 functions in the same molecular pathway as CHC during metaphase in an endocytic-independent manner to stabilise the mitotic spindle for chromosome alignment and subsequent equal segregation of the genome. SNX9 lies downstream of Aurora A activation, but upstream of CHC and TACC3 by contributing to their recruitment to the mitotic spindle, whereby SNX9 recruits CHC, which subsequently recruits TACC3. This provides new insight into the mechanistic pathway of how CHC contributes to chromosome congression by revealing SNX9 as a key mediator of this pathway.

The role of clathrin at the mitotic spindle is dependent on its trimerisation and interaction with the phosphorylated form of TACC3 (transforming acidic coiled-coil-containing protein 3) and ch-TOG (colonic, hepatic tumor overexpressed protein) [8,31,35]. Aurora A kinase phosphorylates TACC3 at S558 and this modification is required for TACC3 spindle localization and clathrin binding [32,38]. TACC3 and ch-TOG are well-characterised spindle components with established roles in MT growth and stabilization [39]. ch-TOG is important for MT outgrowth from centrosomes whilst TACC3 can bind MTs and load ch-TOG onto the spindle [33,34,40]. Although there is consensus as to the role of the clathrin/TACC3/ch-TOG complex at the spindle in cross-linking K-fibres for chromosome alignment and subsequent segregation, there is some disagreement regarding the mechanism by which this complex binds MTs and the role that phosphorylation plays in regulating its function. This has largely come about by opposing reports using siRNA targeting clathrin and TACC3 demonstrating that depletion of one of these proteins causes a reduction in the spindle localization of the other. Thus, there is debate around which protein recruits the other. Two models of how the clathrin/TACC3/ch-TOG complex is recruited to the spindle have been proposed: (1) *The* ‘clathrin recruits TACC3’ *model*. Clathrin is initially recruited to MTs by an unknown protein. Phosphorylated TACC3 binds clathrin and is therefore recruited to the spindle. TACC3 also binds ch-TOG and the MT polymerisation activity of ch-TOG enhances spindle stability [31,35]. *(2) The inter-MT bridge model*. Aurora A-phosphorylated TACC3 is the initial recruitment factor. Clathrin can bind to TACC3/ch-TOG complexes, which may be located on adjacent MTs allowing clathrin to cross-link MTs. The resulting clathrin/TACC3/ch-TOG complex is more stable owing to multiple interactions and therefore accumulates on the spindle [8].

Clathrin does not bind MTs directly, suggesting that it is tethered to the mitotic spindle via protein–protein interactions. Two potential binding partners required for clathrin spindle localisation are B-Myb, a member of the vertebrate Myb family of transcription factors [41] and cyclin G-associated kinase (GAK) [42]. Neither are enriched at the spindle and thus have indirect roles in clathrin spindle localization. B-Myb forms a complex with clathrin and filamin during metaphase and this complex is thought to be important for ferrying clathrin to the spindle. Similarly, reduction in the amount of free clathrin that is able to bind the spindle may be the result of a block in vesicle uncoating in GAK-depleted cells [43,44]. These findings suggest that a centrosome/spindle associated protein is likely to contribute more directly to clathrin spindle recruitment. One such protein is TACC3 and this supports the inter-MT bridge model. However in this study, we identify SNX9 as another such protein, which supports the clathrin recruits TACC3 model. In support of this idea, SNX9 arrives at the mitotic spindle prior to CHC as it is enriched at the spindle poles during prometaphase [16], whereas CHC is not enriched here until metaphase [4]. In contrast to CHC, SNX9 no longer locates to the spindle pole during metaphase and instead redistributes throughout the cytoplasm. It is possible that SNX9 is involved in establishing a pool of CHC at the spindle early on to aid in establishing the mitotic spindle and once initiated SNX9 is no longer required. Other proteins that also contribute to CHC spindle localisation, such as B-Myb [41], TACC3 [8] and GAK [42], may then be involved in maintaining its localisation here. Thus, we propose the following revised mechanistic model of clathrin recruitment that incorporates SNX9 and aspects of both models: i) SNX9 is recruited to the mitotic centrosome during prometaphase (possibly regulated by phosphorylation) where it is involved in the initial recruitment and loading of the clathrin/TACC3/ch-TOG complex onto the spindle as well as MT nucleation to establish the mitotic spindle. ii) SNX9 is displaced from the centrosome and resides in the cytoplasm. ch-TOG binds the plus-ends of nucleated MTs allowing the clathrin/TACC3/ch-TOG complex to travel away from the centrosome during MT polymerisation and mitotic spindle formation. iii) Clathrin is further recruited to the spindle via additional mechanisms, possibly involving TACC3, B-Myb and GAK, further stabilising K-fibre bundles by forming cross-linking bridges.

The metaphase role of SNX9 is dependent on its direct interaction with CHC via its LC domain. The clathrin-binding motifs in the LC domain of SNX9 resemble the non-classical clathrin-binding motifs of amphiphysin that are proposed to bind to the N-terminal β-propeller domain (terminal domain) of clathrin [45]. The terminal domain is responsible for CHC mitotic spindle localization [4]. Given SNX9 does not contain a MT binding region, the SNX9-CHC-TACC3 complex is likely to be tethered to the mitotic spindle via an as yet unidentified binding protein that is capable of binding MTs. The SH3 domain of SNX9 binds many proline-rich containing proteins and potentially tethers SNX9 to the spindle. One potential candidate is dynII, which binds MTs [46] and locates to the spindle poles during metaphase in its phosphorylated form [47].

Finally, SNX9 itself is known to be phosphorylated [21]. The mitotic function of many endocytic proteins is regulated by post-translational modifications such as phosphorylation [47–49]. For example, we have shown that mitotic exit requires dephosphorylation of the endocytic protein, dynII, at S764 [17,47]. In large-scale mass spectrometry-based proteomic studies SNX9 has been reported to be phosphorylated on six residues [50–56]. The SNX9 phospho-sites S176 and Y177 lie in the LC domain and are in close proximity to the second LC CHC binding site, suggesting that phosphorylation at these sites could regulate clathrin binding. Thus, it is possible that a specific phosphorylated pool of SNX9 is targeted to the centrosome during prometaphase. S176 is also a putative Aurora A consensus site and thus like TACC3, recruitment of SNX9 to the centrosome may be regulated by Aurora-mediated phosphorylation. Alternatively, as the other five SNX9 phosphorylation events occur on tyrosine residues then perhaps SNX9 functions in a parallel pathway to Aurora A to regulate clathrin mitotic function, which is regulated by a tyrosine-dependent kinase. It will be important for future studies to determine the role of phosphorylation in regulating SNX9 function during metaphase.

## Supporting Information

Figure S1 **SNX9 is not required for efficient recruitment of Aurora A to the mitotic spindle during metaphase.** (A) Western blots displaying the knock-down efficiency of the TACC3 siRNA in HeLa cells. At 72-h post-transfection, protein lysates (200µg) were immunoblotted with anti-TACC3. Actin served as a loading control. (B) Representative microscopy images of Aurora A localization in untreated and SNX9, CHC or TACC3-depleted HeLa cells during metaphase. (C) The graph represents the fluorescence intensity ratio of Aurora A at the mitotic spindle over the whole cell. (D) Representative microscopy images illustrating the localization of phospho-Aurora A (red) in untreated and SNX9, CHC or TACC3-depleted HeLa cells during metaphase. (E) The graph shows the ratio of fluorescence intensity of phospho-Aurora A (red) at the mitotic spindle compared to the whole cell. Values represent the mean ± S.E.M. from three independent experiments. DNA was stained with DAPI (blue). n > 30 cells analysed from each experiment. ns, not significant; ***, *p* < 0.001 (One-way ANOVA).
(TIF)

Figure S2 **The LC domain of SNX9 is not required for SNX9-dynII interaction.** (B) GST-SNX9 wild-type (WT), LC1 and LC2 mutants coupled to glutathione-Sepharose were used in pull-down experiments from asynchronous HeLa cells and the effect of the individual mutants on dynII and dynII^S764^ binding were visualised by Western blot with anti-dynII and anti-dynI^S778^ antibodies (Figure 4). The amount of DynII (A) and DynII^S764^ (B) bound to the GST-SNX9 mutants were quantified by densitometry analyses of Western blots (n = 3-4 independent experiments). Data is presented as the relative amount of proteins bound to GST-SNX9 (mean ± S.E.M.) compared to GST-SNX9 WT. ns, not significant (One-way ANOVA).
(TIF)

Figure S3 **Disruption of the SNX9-CHC interaction via the LC domain of SNX9 does not affect receptor-mediated endocytosis.** HeLa cells were transfected with GFP empty vector, or GFP-SNX9 WT, LC1 or LC2 mutants and subjected to an endocytosis assay in which the cellular uptake of Alexa Fluor 594-conjugated Tfn was used as the marker for endocytosis. The graph (mean ± S.E.M. from three independent experiments) shows the quantification of Tfn uptake in interphase cells. ns, not significant (One-way ANOVA).
(TIF)
