# Supplementary figures and images for: Sorting Nexin 9 Recruits Clathrin Heavy Chain to the Mitotic Spindle for Chromosome Alignment and Segregation
Source: PLoS One. 2013 Jul 5;8(7):e68387. doi: 10.1371/journal.pone.0068387 (PMC3702553; doi:10.1371/journal.pone.0068387)

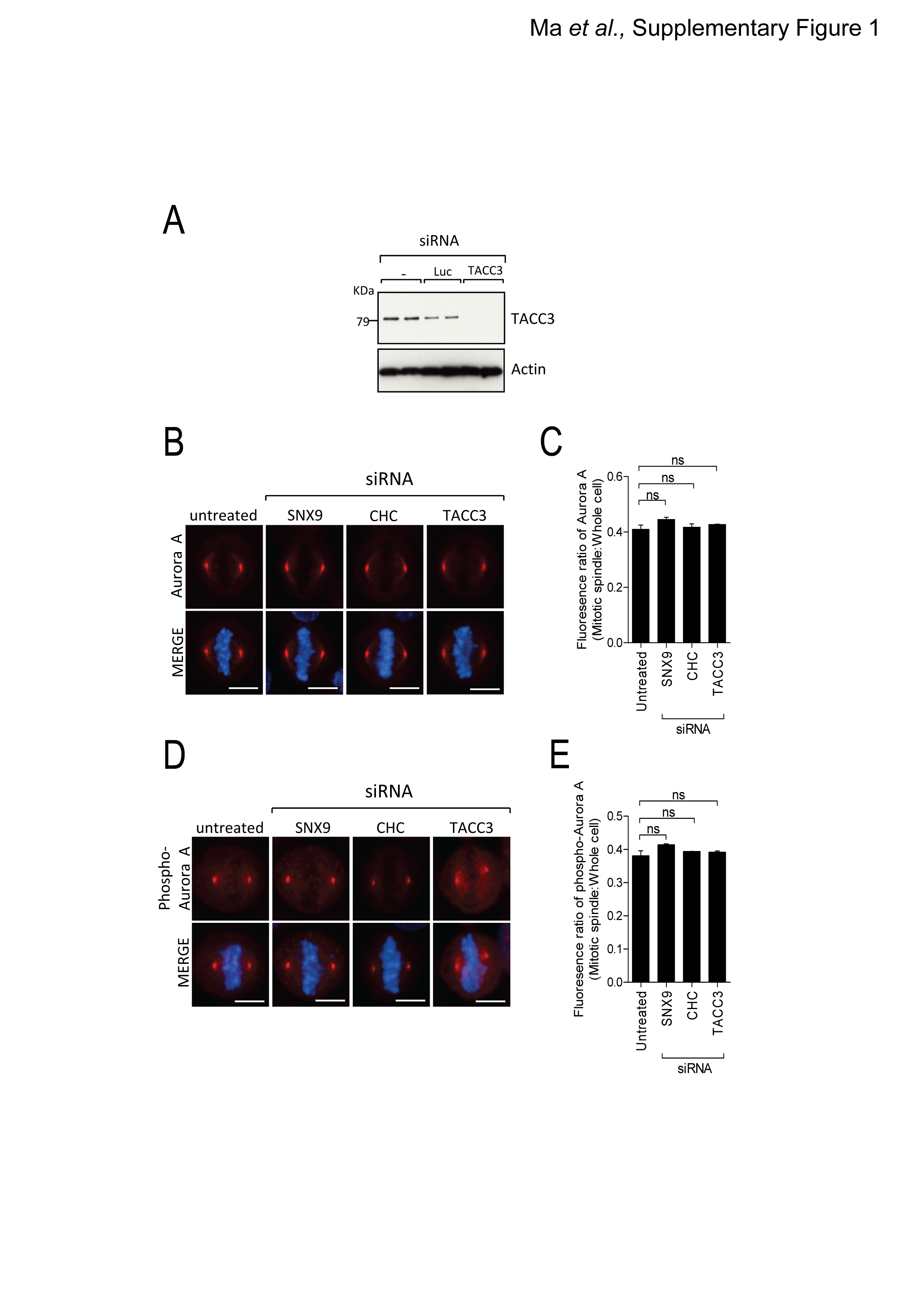

Supplement: Figure S1 — SNX9 is not required for efficient recruitment of Aurora A to the mitotic spindle during metaphase. (A) Western blots displaying the knock-down efficiency of the TACC3 siRNA in HeLa cells. At 72-h post-transfection, protein lysates (200µg) were immunoblotted with anti-TACC3. Actin served as a loading control. (B) Representative microscopy images of Aurora A localization in untreated and SNX9, CHC or TACC3-depleted HeLa cells during metaphase. (C) The graph represents the fluorescence intensity ratio of Aurora A at the mitotic spindle over the whole cell. (D) Representative microscopy images illustrating the localization of phospho-Aurora A (red) in untreated and SNX9, CHC or TACC3-depleted HeLa cells during metaphase. (E) The graph shows the ratio of fluorescence intensity of phospho-Aurora A (red) at the mitotic spindle compared to the whole cell. Values represent the mean ± S.E.M. from three independent experiments. DNA was stained with DAPI (blue). n > 30 cells analysed from each experiment. ns, not significant; ***, p < 0.001 (One-way ANOVA). (TIF) [file pone.0068387.s001.tif]

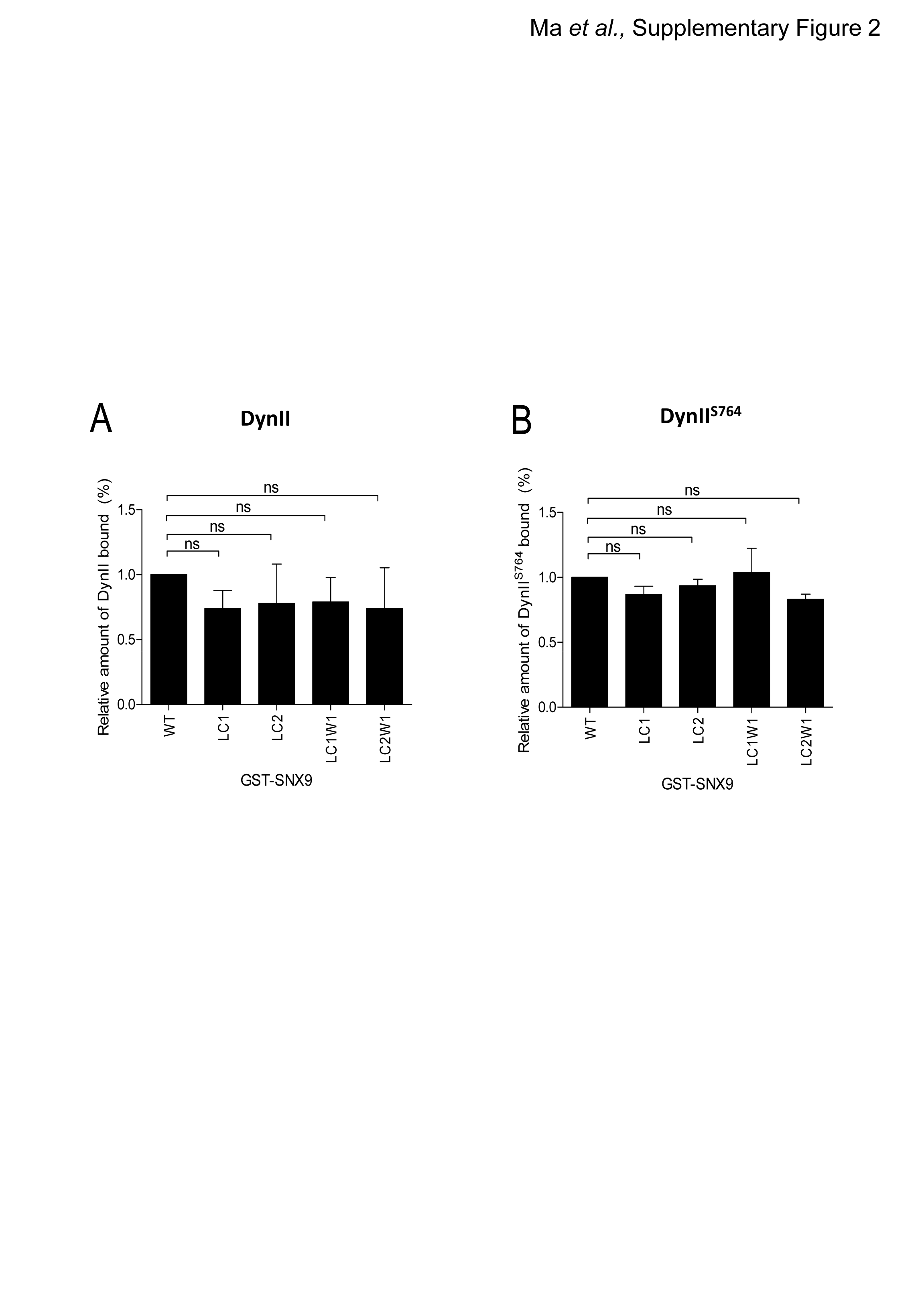

Supplement: Figure S2 — The LC domain of SNX9 is not required for SNX9-dynII interaction. (B) GST-SNX9 wild-type (WT), LC1 and LC2 mutants coupled to glutathione-Sepharose were used in pull-down experiments from asynchronous HeLa cells and the effect of the individual mutants on dynII and dynIIS764 binding were visualised by Western blot with anti-dynII and anti-dynIS778 antibodies (Figure 4). The amount of DynII (A) and DynIIS764 (B) bound to the GST-SNX9 mutants were quantified by densitometry analyses of Western blots (n = 3-4 independent experiments). Data is presented as the relative amount of proteins bound to GST-SNX9 (mean ± S.E.M.) compared to GST-SNX9 WT. ns, not significant (One-way ANOVA). (TIF) [file pone.0068387.s002.tif]

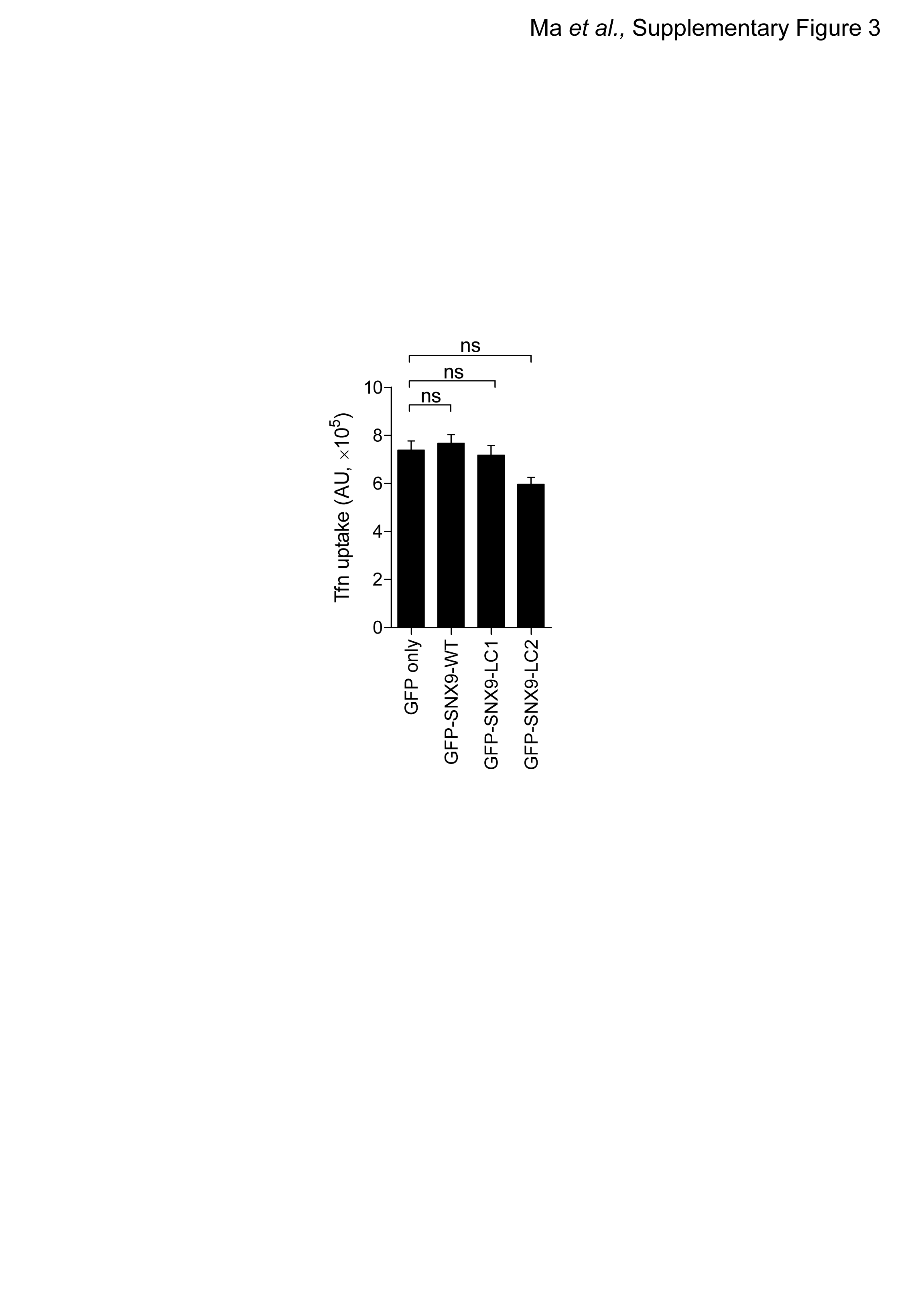

Supplement: Figure S3 — Disruption of the SNX9-CHC interaction via the LC domain of SNX9 does not affect receptor-mediated endocytosis. HeLa cells were transfected with GFP empty vector, or GFP-SNX9 WT, LC1 or LC2 mutants and subjected to an endocytosis assay in which the cellular uptake of Alexa Fluor 594-conjugated Tfn was used as the marker for endocytosis. The graph (mean ± S.E.M. from three independent experiments) shows the quantification of Tfn uptake in interphase cells. ns, not significant (One-way ANOVA). (TIF) [file pone.0068387.s003.tif]
